# Care burden on family caregivers of patients with dementia and affecting factors in China: A systematic review

**DOI:** 10.3389/fpsyt.2022.1004552

**Published:** 2022-12-05

**Authors:** Lan Wang, Yang Zhou, Xiaofeng Fang, Guiyu Qu

**Affiliations:** ^1^School of Nursing, Weifang Medical University, Weifang, China; ^2^Weifang People’s Hospital, Weifang, China

**Keywords:** family caregivers, dementia, care burden, caregiving, systematic review

## Abstract

**Background:**

Dementia is a chronic and progressive illness characterized by severe impairment and high dependencies. Under the influence of Chinese traditional culture, 80% of patients with dementia are watched over at home by family caregivers as primary caregivers. However, long-term care brings formidable burdens to them and reduces the quality of their life. It is necessary to find out the influencing factors of caregivers’ burden.

**Methods:**

A scoping search was conducted on eight electronic databases from 1 January 2010 to 14 June 2022: PubMed, Embase, the Cochrane Library, Web of Science, China National Knowledge Infrastructure, China VIP Database, China Biomedical Literature Database, and Wanfang Data Knowledge Service Platform. Research articles included in this review discussed the factors affecting Chinese dementia family caregivers’ care burden or stress, and the level of care burden was evaluated by a standardized care burden scale.

**Results:**

A total of 1,888 related articles were found and 23 cross-sectional studies were eventually included. After quality assessment, 12 were of good quality and 11 were of fair quality. A total of 32 factors were identified that were associated with caregiver burden, and the results were grouped into three categories: patient, caregiver, and society. The severity of disease, poor self-care ability, neuropsychiatric symptoms, care time, number of helpers, poor health status, economic stress, poor psychological status, social support, and age were reported in many previous studies.

**Conclusion:**

In this review, the factors that affect the caregiver burden for people with dementia were clarified. By identifying these factors, hospitals, decision-makers, and communities can carry out special projects for these populations, provide appropriate assistance, or design corresponding intervention measures to reduce the caregiver burden and improve the quality of care for patients with dementia.

**Systematic review registration:**

[https://www.crd.york.ac.uk/PROSPERO/], identifier [CRD42022347816].

## Introduction

Dementia has increasingly become a universal public health issue (1). In China, the incidence rate of dementia in elderly people from 60 to 69 years is 2.9%, while it is 31.9% for those above 90 years (2). With the aging of China’s population, the number of elderly patients with dementia has risen (3). According to the latest statistics, in 2019, over 15.33 million Chinese people were suffering from dementia and the number is expected to be 45.33 million in 2050 (4). Dementia is a chronic and progressive illness characterized by severe impairment and high dependencies (5, 6). Patients with moderate-to-severe degrees of dementia also need full-time care; caregivers’ duty mainly includes daily tasks, meeting emotional needs, and paying bills. Therefore, caregivers are expected to take more responsibilities as the condition of patients with dementia deteriorates.

Home care is essential in China and around the world since people with dementia and Alzheimer’s disease are on the rise. Especially, in China, Filial piety (or Xiao) in Confucianism values is the core of Chinese traditional culture and an important part of family ethics, and it mandates adults to respect and take care of their parents (7). Traditionally, only childless or poor elderly people enter public care, and Chinese elder individuals also feel ostracized and prejudiced against entering nursing homes (8). The influence of the filial piety notion forces the relatives of the elderly to be more willing to care for the elderly at home, and 80% of patients with dementia are therefore watched over at home with family caregivers as primary caregivers (5).

However, studies have demonstrated that dementia exerts a heavy burden on the family. Providing care for patients with dementia can be a daunting task for family caregivers and also an uncomfortable experience, involving physical, mental, financial, and social aspects (9, 10). Caring for a person with dementia presents challenges that are different from other caregiving situations, mainly because the family can feel bereft since patients are unable to communicate effectively, express love, or even forget everything, which can add to their internal pain and induce mental burnout (11). In addition, the level of depression and mental health issues among family caregivers was significantly higher than among other caregivers for chronic diseases according to the findings of recent studies (12, 13). Given the high prevalence of dementia and the considerable effect of care burden on caregivers’ health, it is necessary to better understand the influencing factors of caregivers’ burden.

Unfortunately, although a growing number of studies have concentrated on the family care burden for dementia in China, some factors remain controversial. For example, Wang et al. (14) concluded that increasing helpers would not reduce the caregiving burden, but Bai (15) came to the opposite conclusion; He et al. (16) believed that financial burden was the most important factor affecting the caregiver, while Huang et al. (17) believed that the number of nocturnal awakenings had the greatest impact. Moreover, a systematic review (18) of the care burden of patients with dementia in Turkey identified that older age patients increased caregiver burden; nevertheless, some studies in China were contradictory. Therefore, our research aims to systematically identify factors and the existing problems and to help the healthcare system to lay down intervention schemes based on our review; the review will also provide evidence for the development of dementia care services in other countries, especially those similar to China in culture and social landscape.

## Materials and methods

This review was performed in accordance with the Preferred Reporting Items for Systematic Reviews and Meta-Analyses Statement (PRISMA) guideline. The methodology of this systematic review has been published in Prospero Platform (CRD42022347816), and we have recently revised the protocol to improve the rigor of the method description.

### Search strategy

A scoping search for studies published from 1 January 2010 to 14 June 2022 was conducted on eight electronic databases: PubMed, Embase, the Cochrane Library, Web of Science, China National Knowledge Infrastructure (CNKI), China VIP Database (VIP), China Biomedical Literature Database (CBM), and Wanfang Data Knowledge Service Platform. The search strategy employed MeSH terms and keywords to identify the potential studies. Search terms for the review were (“dementia” OR “Alzheimer’s disease” OR “dementia*” OR “Alzheimer*” OR “vascular dementia” OR “senile dementia” OR “mixed dementia” OR “Senile Paranoid Dementia” OR “Amentia*”) AND (“caregiver” OR “caregiv*” OR “carer*” OR “family caregiver” OR “informal caregiver” OR “home caregiver”) AND (“burden” OR “strain” OR “stress” OR “distress” OR “suffer” OR “overload”) AND (“Chinese” OR “China”). The search strategy was jointly determined by two reviewers, and the reference list of all selected studies was manually retrieved to find out eligible articles.

### Eligibility criteria

The eligibility criteria for review were based on the “PICOS” principles. Populations: family caregivers of dementia aged 18 years and above in China, including spouses, children, siblings, friends, or other relatives; outcomes: affecting factors of family caregivers’ care burden or stress and the level of care burden were measured at any course of dementia by a standardized care burden scale; and study design: observational studies, such as descriptive studies, cohort studies, and cross-sectional studies. Considering the outcomes to be explored, intervention and control are not applicable here. Furthermore, these studies were published in Chinese or English. The exclusion criteria were health professionals or salaried caregivers; reviews, case reports, conference abstracts, trial protocols, non-peer-reviewed articles, and original studies without full texts or reliable data; and studies were also excluded if the quality appraisal score was 0–4.

### Study selection and data extraction

The searched studies were first imported in EndNoteX9.1, then duplicate studies were deleted, and the title and abstract of articles were screened. Finally, the full text was read and the studies meeting the inclusion criteria were included. Two reviewers (LW and YZ) independently screened and extracted literature and cross-checked it, and any disagreements were discussed by LW and YZ to reach a consensus, or differences of opinions were eliminated by asking for a third party’s suggestion. The following characteristics of included studies were extracted: author, publication year, study design, sample size, caring relationship, co-residence, research tools, burden/stress score, and influencing factors.

### Quality assessment

Two reviewers (LW and YZ) independently used the modified Newcastle–Ottawa Scale (NOS) (19, 20) to assess the methodological quality of the included studies. This tool is adapted for cross-sectional studies and contains seven questions in three groups, including representativeness of the sample, sample size adequacy, non-respondents, ascertainment of the exposure (risk factor), comparability in different outcome groups based on the study design or analysis, assessment of the outcome, and statistical test. All items are one point, except for the fifth, which is two points. Studies that received seven-eight and five-six points were considered good and fair quality, respectively. In addition, any disagreements would be solved by the third reviewer (XFF).

### Analyses

This review used qualitative analysis to systematically summarize and describe the information and results of the included studies. The main contents include (1) basic information about the studies; and (2) classification and description of the relevant results based on different influencing factors.

## Results

### Search results and quality assessment

**FIGURE 1:**
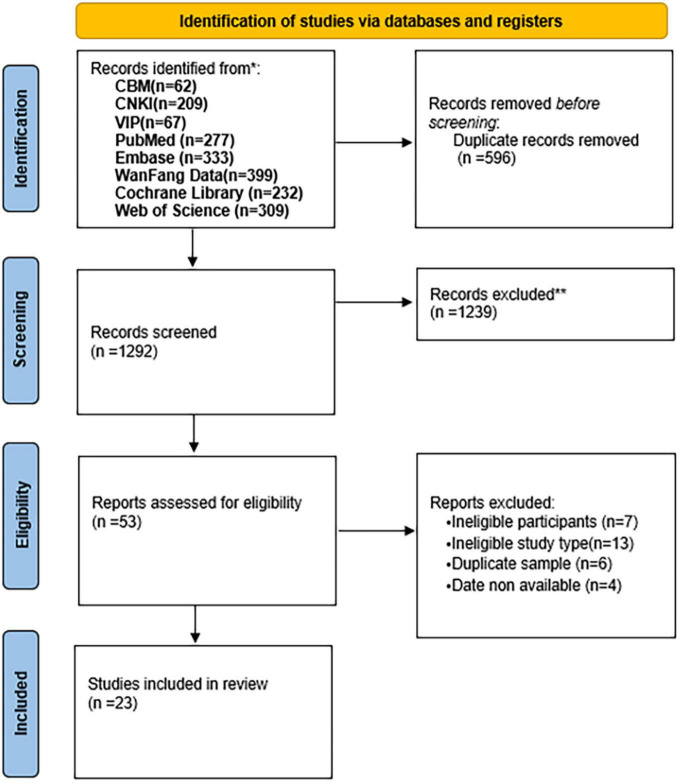
Preferred reporting items for systematic reviews and meta-analyses statement (PRISMA) study flowchart of study selection.

A total of 1,888 potentially related articles were found. Among them, 596 duplicated studies were removed, 1,239 were also excluded after reading the title and abstract, 53 studies were chosen for full-text screening, and 23 cross-sectional studies were eventually included in the review. Figure 1 shows the PRISMA study flowchart of study selection (Figure 1). After quality assessment, 12 studies were of good quality and 11 were of fair quality. The result is shown in Supplementary Table 1.

### Characteristics of included studies

Supplementary Table 2 depicts the characteristics of the included studies. Of the 23 studies, 8 (14, 21–27) (34.8%) were in English and 15 (15–17, 28–39) (65.2%) were in Chinese. In addition, 14 studies (14–16, 21, 23, 27–29, 32, 34–39) (60.9%) employed Caregiver Burden Inventory (CBI), 7 (17, 22, 24–26, 31, 33) (30.4%) employed Zarit Burden Interview (ZBI), 1 (30) (4.3%) employed Family Burden Scale (FBS), and 1 (15) (4.3%) employed Perceived Stress Scale (PSS). This review included 4,076 participants, and the sample sizes ranged from 94 to 335. Studies (15, 30, 33, 37) have indicated that 70.3–100% of family caregivers bear the burden of care. In addition, primary family caregivers in 14 studies (14, 16, 22, 23, 26, 28–30, 32–37) (60.9%) were offspring or other relatives and in six studies (15, 24, 25, 27, 31, 38) (26.1%) were spouses, but three studies (17, 21, 39) (13%) were failed to mention the proportion of caregivers.

### Description of associated factors of caregiver burden

A total of 32 factors were identified that were associated with caregiver burden. These factors have been analyzed and grouped into three categories based on their characteristics, including patient, caregiver, and social factors (Table 1). The detailed description is as follows.

**TABLE 1:** Summary of factors associated with caregiver burden.

	References	Participant number
**Patient factors**		
**Demographics**		
Retirement pension	3 (29, 30, 37)	534
Ounger age	3 (14, 30, 37)	502
Low eucation level	1 (37)	160
Religious belief	1 (39)	105
**Disease-related**		
Severity of dementia	7 (14, 15, 24, 26, 30, 34, 36)	1,751
Poor self-care ability	7 (24, 25, 28, 32, 34, 35, 37)	1,525
Neuropsychiatric symptoms	6 (14, 22, 25, 30, 32, 33)	1,060
Low cognitive function	2 (23, 27)	462
Memory behavior problems	2 (22, 36)	436
Low quality of life	1 (34)	335
Physical dysfunction	1 (28)	152
Disease type	1 (17)	97
**Caregiver factors**		
**Demographics**		
Economic stress	6 (16, 25, 31, 32, 37, 38)	1,061
Caregiver’s age	3 (17, 25, 36)	1,001
Unemployed or retired caregiver	3 (14, 26, 30)	642
Female caregivers	3 (14, 22, 37)	454
Low education level	2 (35, 38)	270
Divorced or widowed caregiver	1 (38)	176
Religious belief	1 (21)	152
**Caregiving-related**		
Care time	13 (14–17, 21–23, 28–31, 35, 38)	2,064
Number of helpers	5 (15, 28, 31, 33, 35)	671
Cohabiting with the patient	3 (14, 17, 32)	532
Role perceptions	3 (15, 21, 35)	417
Number of nocturnal awakenings	2 (17, 37)	393
Positive aspects of caregiving	2 (23, 27)	277
Disease awareness	2 (28, 35)	246
Feel discrimination	1 (39)	105
Undertake household duties	1 (16)	97
**Health status**		
Poor health status	7 (14, 15, 24, 30, 31, 33, 39)	1,181
Poor psychologic status	4 (15, 24, 29, 37)	824
**Social factors**		
Social support	3 (14, 23, 27)	429
Usage of community service	1 (27)	109

#### Patient factors

Notably, 12 patient factors were identified, and the severity of dementia, poor self-care ability, and neuropsychiatric symptoms were the three most frequently mentioned patient factors. In terms of patient demographics, studies reported that patients with younger ages (14, 30, 37), less or no retirement pension (29, 30, 37), low education (37), and religious belief (39) were associated with higher care burdens. In disease-related factors, studies indicated that patients with higher severity of dementia (14, 15, 24, 26, 30, 34, 36), poor self-care ability (24, 25, 28, 32, 34, 35, 37), neuropsychiatric symptoms (14, 22, 25, 30, 32, 33), low cognitive function (23, 27), memory behavior problems (22, 36), Alzheimer’s disease (17), and physical dysfunction (28) were risk factors for increased caregiver burden. In addition, one study (34) found that low patients’ quality of life is an independent influencing factor for increased burden.

#### Caregiver factors

A total of 18 caregiver factors were found, of which the number of helpers and positive aspects of caregiving were protective factors, meaning that caregivers would have less caregiving burden if they have helpers (15, 28, 31, 33, 35) or a higher degree of positive aspects of caregiving (23, 27). In terms of caregiver demographics, family caregivers with economic stress (16, 25, 31, 32, 37, 38), female (14, 22, 37), unemployed or retired (14, 26, 30), low education level (35, 38), divorced or widowed (38), and religious belief (21) experienced higher levels of care burden. Caregivers who had poor health status (14, 15, 24, 30, 31, 33, 39), cohabiting with the patients (14, 17, 25, 32), poor role perceptions (15, 21, 35), a high number of nocturnal awakenings (17, 37), poor disease awareness (28, 35), undertake household duties (16), and feel discrimination (39) were associated with higher degrees of care burden. Of the 23 studies, 13 studies (14–17, 21–23, 28–31, 35, 38) found a significant and negative relationship between family caregivers and care time. Notably, four studies reported that psychological status was closely related to caregiver burden, especially depression (24, 29) and anxiety (15, 29, 37). the age of the caregiver also has an impact on the care burden. One study (17) showed that older caregivers experienced more burden, whereas another two studies (25, 36) showed that younger people experienced more burden.

#### Social factors

A total of two social factors were identified, including social support and usage of community service. While three studies (14, 23, 27) showed that social support was negatively related to care burden. Liu et al. (27) found that caregivers would have a higher level of burden after using community service.

## Discussion

This review identified and consolidated many different variables that affect the burden on Chinese caregivers for patients with dementia, and it is possible to achieve some general conclusions from the results. Among the factors, the severity of dementia, poor self-care ability, neuropsychiatric symptoms, economic stress, care time, number of helpers, poor health status, and poor psychological status could be found in many studies.

For patient factors, the severity of dementia, poor self-care ability, and neuropsychiatric symptoms were the most burdensome to caregivers, which were consistent with the previous research (9, 18, 40). As the severity of dementia increases, the need and difficulty in taking care of patients also increase. Patients with severe dementia presented poor self-care ability and mobility; thus, family members need more time and energy for intensive care (14, 15, 24, 26, 30, 34, 36). Chinese healthcare providers have implemented the continuum of care for dementia to raise awareness, risk assessment and screening, and early diagnosis of dementia; however, low diagnosis rates and delays in seeking care for patients with dementia remain a significant concern (3, 33, 41). Thus, more efforts are needed to enhance the early detection of the disease.

Furthermore, we also found that there was a strong positive correlation between neuropsychiatric symptoms of patients with dementia and caregivers’ burden (14, 22, 25, 30, 32, 33), especially when patients had symptoms such as agitation, irritability, abnormal motor behavior, depression, and hallucinations (25, 42). These symptoms would not only hinder the treatment and increase the difficulty of care (30, 33) but also make caregivers feel helpless and sad and even contribute to anxiety and depression when they faced unfamiliar family members (11, 32). In a comparison between Australia and China, Xiao et al. (5) concluded that the prevalence of behavioral and psychological symptoms of dementia (BPSD) among Chinese patients is higher than the 61–88% prevalence in Australia, which may be attributed to underdeveloped geriatric care facilities and lack of behavioral management and services for dementia, and Wang et al. (14) also showed the same result. Strong establishment and improvement of caregiver rehabilitation programs and dementia behavior management services will be needed in the future to reduce neuropsychiatric symptoms of dementia (14, 30, 33).

It is noteworthy that the time spent providing care was considered an important factor of caregiver burden, as this was not strongly emphasized in the previous systematic reviews (18, 40). Wang et al. (14) study showed that Chinese caregivers spent an average of 127.6 h per week on care, which is significantly more intensive than the 27.1 h per caregiver per week reported in developed countries (12). This can be explained by the fact that cultural awareness of caregiving obligations may augment the time burden felt by caregivers (26). Caregivers are influenced by filial piety and view taking care of family members as their responsibility. Moreover, they believe that it is unacceptable to share family affairs with outsiders, and the fear of discrimination, if others know about it, may prevent them from actively seeking others’ help (14, 27, 35, 39). However, several studies have consistently stated that if other helpers were available, the burden on family caregivers would be significantly reduced (15, 28, 31, 33, 35, 43). Therefore, in the future, we should draw on the successful experiences of other countries to establish community-based day-care centers, short-term care facilities, and respite service measures such as providing in-home care to reduce direct caregiver time (30, 31, 35). In addition, the number of nocturnal awakenings also indirectly increases the caregiver’s care time and prevents caregivers from meeting their sleep needs (17, 37).

The result of this review was consistent with Adana et al. (18) and Chiao et al. (40), indicating that female caregivers had a higher care burden (14, 22, 37). A previous study demonstrated that female caregivers performed caregiving tasks 2.5 times more than male caregivers (44), which may be in line with the traditional Chinese saying of men rules outside and women rules inside (nan zhu wai and nu zhu nei). Women always play the role of primary caregivers for all the family members with multiple household tasks (18, 22, 37). Moreover, female caregivers pay more attention to the quality of care and the relationship with patients, devote more energy and time, and tend to experience intense guilt and stress (45), resulting in both physiological and psychological burdens. However, a systematic review (46) emphasized that male caregivers experienced a higher care burden because of a lack of social readiness as they faced role changes. Men are also likely to become primary caregivers in the future (47), but less is known about the condition and needs of male caregivers (48). We, therefore, need more research to be conducted in the future to explore how male caregivers cope with their caregiver role.

Some studies identified that caregivers with poor health and financial issue are more stressed. When caregivers suffer from diseases, they usually cannot take care of themselves through tedious care. Coupled with lasting mental stress, caregivers are under great pressure, which will formulate a vicious circle and aggravate their illness (15, 30, 31, 33, 39). The median monthly direct medical cost of caregiver expenditures reported in He’s study was 600 RMB and 78.4% of caregivers felt financial stress (16), and one study has stated that patients with severe dementia spent nearly two times as much on annual expenses as those patients with mild dementia (10). Thus, more severe dementia indicates a more severe financial burden for caregivers. To ease the financial burden of caregivers, Sweden and the United States have offered caregiving as a formal profession and paid for full-time caregivers, and Canada, Sweden, and the United Kingdom have offered tax benefits for caregivers, while China does not have care subsidies directly for family caregivers (49, 50). Furthermore, dementia drug costs have been integrated into health insurance in China, but some problems such as few illness types and little money for reimbursement still exist, and families still have to bear most of the medical bills (16, 32, 37).

Also, some studies have reported that caregivers were significantly more vulnerable to suffering from anxiety and depression (15, 24, 29, 37, 51, 52). The psychological burden has become the most important aspect (29, 52), and this may be related to the fact that the caregiver’s life is mainly focused on caring for the patient and has no time for personal life, employment, and social life (37), which creates a strong role conflict. In addition, the high cost of treatment, the patient’s BPSD symptoms, and the poor health status of caregivers can have a negative impact on the caregiver’s psychology (15, 29, 37). Meanwhile, lasting negative emotions increased the risk of patient abuse among family caregivers (53). Consequently, caregivers need psychological support to help them develop reasonable emotional expressions, enhance their ability to seek help and solve problems, and prevent and reduce the occurrence of psychological problems.

The effect of age on the burden remains controversial. Two studies (30, 37) revealed that caregivers perceived the heaviest burden when patients were less than 70 years of age. Younger patients commonly undertake many family responsibilities and higher expectations, so the absence of patients’ roles could cause higher dissatisfaction; meanwhile, diagnosed patients usually felt embarrassed so they are likely to hide their illness and delay therapy. However, Adana et al. (18) and Thyrian et al. (54) found contradictory results and suggested that disease and advanced age resulted in this finding. The age of the caregiver also influences the care burden. One study (17) demonstrated that older caregivers have greater physiological and psychological burdens, while other studies (14, 18, 25, 36, 46) hold the opposite view. Future large-scale and high-quality longitudinal studies should be undertaken to authenticate the age factor.

Low education level (35, 38) and poor disease awareness (28, 35) were associated with a higher caregiving burden. Caregivers with low education levels are less knowledgeable about dementia disease and are prone to mood swings when faced with disease-related events such as the prognosis of recovery and deterioration of the disease, resulting in a heavier psychological burden (35, 38). Compared to 40% of dementia caregivers in the United States who have a college degree or higher (12), caregivers in China have a relatively low level of education (55). Furthermore, 49.0% of Chinese caregivers had disease awareness significantly lower than the overall level of 56.5%, and only 35.7% of patients had disease awareness (56). Therefore, caregivers need the training to enhance their knowledge on disease and caregiving skills. It is noticeable that there are only 6.25% of the 752 Chinese dementia caregivers had received caregiving training according to a questionnaire result (57).

The current review shows that social support is a protective factor that can lessen the caregiving burden (14, 23, 27). Social support is a buffer to regulate life events and psychological stress (58), which is beneficial to physical and mental health. When receiving more supportive services from family, friends, occupation, and community, caregivers can markedly reduce their burden (23, 27, 58). Long-term care insurance policy has been implemented in 15 pilot cities in China since 2016, and one study found that patients covered by long-term care insurance were only required to pay 10% of the cost for the services they received (59), which significantly reduces the financial burden on families. However, many patients with dementia were not covered. The policy was still not powerful enough to meet the need of the patient family (60). In some developed countries, daily care has been jointly undertaken by the home and community. However, the community does not work well due to the sociocultural background in China, the lack of professional dementia care services, and insurance support (26). Thus, a hospital–community–family-integrated social support model can be established to assist caregivers in all aspects (61).

We critically analyze the caregiver burden of home-based families in the Chinese cultural context and identify some problems, highlighting the way to future construction. In addition, our review can also bring inspiration to some developing countries to help alleviate their caregiver burden. There are also several limitations to this study. First, the included articles are cross-sectional studies with poor causation. Second, the study used different scales to measure the caregiving burden, which may differ in the interpretation of the results, and finally, although we included two studies from Hong Kong and Taiwan, we did not conduct a detailed analysis because their policies and circumstances are different from those of the mainland.

In summary, family caregivers of patients with dementia generally have burdens, and 32 factors were identified that were associated with caregiver burden. Among them, the severity of disease, poor self-care ability, neuropsychiatric symptoms, care time, poor health status, economic stress, poor psychological status, and social support were considered as main factors, but there was no conclusion on age. By identifying these factors, hospitals, decision-makers, and communities can carry out special projects for these populations, to provide appropriate assistance, or design corresponding intervention measures to reduce the caregiver burden and improve the quality of care for patients with dementia.

## Data availability statement

The original contributions presented in this study are included in the article/Supplementary material, further inquiries can be directed to the corresponding author.

## Author contributions

LW and YZ conducted the study, carried out the statistical analysis, and wrote the manuscript. XF supported the development of the study methodology and reviewed the manuscript. GQ supported the writing of the manuscript and supervised the whole process. All authors contributed to the article and approved the submitted version.
