# Supplementary material for: Care burden on family caregivers of patients with dementia and affecting factors in China: A systematic review
Source: Front Psychiatry. 2022 Dec 5;13:1004552. doi: 10.3389/fpsyt.2022.1004552 (PMC9760850; doi:10.3389/fpsyt.2022.1004552)
Supplement: Supplementary file 1 [file Table_1.DOCX]

**Supplementary table 1. Quality assessment of studies.**

| **Study** | **Selection** | | | | **Comparability** | **Outcome** | | **Score** | **Rank** |
| --- | --- | --- | --- | --- | --- | --- | --- | --- | --- |
|  | **1** | **2** | **3** | **4** | **5** | **6** | **7** |  |  |
| **Jing Wang(14)** | 0 | 1 | 1 | 0 | 1 | 1 | 1 | 5 | Fair |
| **Xiulan Bai(15)** | 1 | 1 | 1 | 1 | 2 | 1 | 1 | 8 | Good |
| **Sinmin He(16)** | 1 | 0 | 1 | 1 | 2 | 1 | 1 | 7 | Good |
| **Wei Huang(17)** | 1 | 1 | 1 | 1 | 1 | 1 | 1 | 7 | Good |
| **Hong Li(21)** | 1 | 0 | 1 | 1 | 2 | 1 | 1 | 7 | Good |
| **Sheung-Tak Cheng(22)** | 1 | 0 | 1 | 1 | 1 | 1 | 1 | 6 | Fair |
| **Hongmei Yu(23)** | 1 | 0 | 1 | 0 | 1 | 1 | 1 | 5 | Fair |
| **Shuai Liu(24)** | 1 | 0 | 1 | 1 | 2 | 1 | 1 | 7 | Good |
| **Chia-Fen Tsai(25)** | 1 | 0 | 1 | 1 | 1 | 1 | 1 | 6 | Fair |
| **Xuejun Yin(26)** | 1 | 0 | 1 | 1 | 1 | 1 | 1 | 6 | Fair |
| **Zhijian Liu(27)** | 1 | 1 | 1 | 1 | 2 | 1 | 1 | 8 | Good |
| **Honghui Zhang(28)** | 1 | 0 | 1 | 1 | 1 | 1 | 1 | 6 | Fair |
| **Jiao Luo(29)** | 1 | 1 | 1 | 1 | 2 | 1 | 1 | 8 | Good |
| **Yan Ding(30)** | 1 | 1 | 1 | 1 | 1 | 1 | 1 | 7 | Good |
| **Fen Jiang(31)** | 1 | 0 | 1 | 1 | 1 | 1 | 1 | 6 | Fair |
| **Jian Zou(32)** | 1 | 1 | 1 | 1 | 1 | 1 | 1 | 7 | Good |
| **Xing Wu(33)** | 1 | 0 | 1 | 1 | 1 | 1 | 1 | 6 | Fair |
| **Jianjun Yin(34)** | 1 | 0 | 1 | 1 | 1 | 1 | 1 | 6 | Fair |
| **Meili Yang(35)** | 1 | 0 | 1 | 1 | 2 | 1 | 1 | 7 | Good |
| **Masami Fukuda(36)** | 1 | 1 | 1 | 1 | 2 | 1 | 1 | 8 | Good |
| **Fang Jin(37)** | 1 | 1 | 1 | 1 | 1 | 1 | 1 | 7 | Good |
| **Jun Jin(38)** | 1 | 0 | 1 | 1 | 1 | 1 | 1 | 6 | Fair |
| **Yan Zhang(39)** | 1 | 0 | 1 | 0 | 1 | 1 | 1 | 5 | Fair |

Note: 1—representativeness of the sample; 2—sample size adequacy; 3—non-respondents; 4—ascertainment of the exposure (risk factor); 5—comparability in different outcome groups based on the study design or analysis; 6—assessment of the outcome; 7—statistical test
