# Supplementary material for: Care burden on family caregivers of patients with dementia and affecting factors in China: A systematic review
Source: Front Psychiatry. 2022 Dec 5;13:1004552. doi: 10.3389/fpsyt.2022.1004552 (PMC9760850; doi:10.3389/fpsyt.2022.1004552)
Supplement: Supplementary file 2 [file Table_2.docx]

**Supplementary table 2**: General description of studies included in the review.

| **Author (Year)** | **Study Design** | **Number of**  **caregivers** | **Caring relationship** | **Co-residence** | **Study location** | **Caregiver burden tools** | **Burden score** | **Quality assessment** |
| --- | --- | --- | --- | --- | --- | --- | --- | --- |
| **Jing Wang(14)**  **2014** | cross-sectional study | 152 | Spouses (36.2%)  Non-spouses (63.8%) | 117/35(77%) | Community | CBI | Not mentioned | 5 |
| **Xiulan Bai(15)**  **2021** | cross-sectional study | 171 | Spouses (59.6%)  Children (35.1%)  Other relatives (5.3%) | Not mentioned | Hospital | PSS | 31.19±6. 91 | 8 |
| **Sinmin He(16)**  **2020** | cross-sectional study | 97 | Spouses (16.49%)  Children (69.07%)  Other relatives (14.44%) | 39/58(40.2%) | Hospital | CBI | 40.43±20.05 | 7 |
| **Wei Huang(17)**  **2016** | cross-sectional study | 233 | Not mentioned | 181/48(77.7%) | Hospital | ZBI | 57.56±13.45 | 7 |
| **Hong Li(21)**  **2012** | cross-sectional study | 152 | Not mentioned | 137/15(90.1%) | Patients home | CBI | 24.32±19.35 | 7 |
| **Sheung-Tak Cheng(22)**  **2013** | cross-sectional study | 142 | Spouses (32%%)  Children (59%%)  Other relatives (9%) | 79% | Hospital,  Association  Community | ZBI | 24.46±14.83 | 6 |
| **Hongmei Yu(23)**  **2015** | cross-sectional study | 168 | Spouses (42.3%)  Children (45.8%)  Other relatives (11.9%) | Not mentioned | Hospital  Community | CBI | 47.5±17.6 | 5 |
| **Shuai Liu(24)**  **2017** | cross-sectional study | 309 | Spouses (58.9%)  Children (36.5%)  Other relatives (4.5%) | 254/55(82.2%) | Hospital | ZBI | 12.2±13.2 | 7 |
| **Chia-Fen Tsai(25)**  **2021** | cross-sectional study | 328 | Spouses (33.24%)  Children (49.34%)  Other relatives (17.42%) | 300/38(91.5%) | Hospital | ZBI | 26.7±18.1 | 6 |
| **Xuejun Yin(26)**  **2021** | cross-sectional study | 300 | Spouses (18.33%)  Children (71.67%)  Other relatives (10%) | Not mentioned | Hospital | ZBI | 43.05±13.42 | 6 |
| **Zhijian Liu(27)**  **2022** | cross-sectional study | 109 | Spouses (50.6%)  Children (36.7%)  Other relatives (12.7%) | Not mentioned | Community | CBI | 65.92±16.74 | 8 |
| **Honghui Zhang(28)**  **2010** | cross-sectional study | 152 | Spouses (33.24%)  Children (49.34%)  Other relatives (17.42%) | Not mentioned | Patients home | CBI | Not mentioned | 6 |
| **Jiao Luo(29)**  **2011** | cross-sectional study | 184 | Spouses (33.24%)  Children (49.34%)  Other relatives (17.42%) | 151/33(82.1%) | Hospital  Community | CBI | 45.30±12.76 | 8 |
| **Yan Ding(30)**  **2014** | cross-sectional study | 190 | Spouses (45.8%)  Children (47.9%)  Other relatives (6.3%)) | Not mentioned | Hospital  Community | FBS | 26.69±9.8 | 7 |
| **Fen Jiang(31)**  **2014** | cross-sectional study | 153 | Spouses (56.2%)  Children (35.9%)  Other relatives (7.9%) | 95/58(62.1%) | Hospital | ZBI | 38.25±14.29 | 6 |
| **Jian Zou(32)**  **2014** | cross-sectional study | 147 | Spouses (42.9%)  Children (42.9%)  Other relatives (14.2%) | 100/47(68%) | Hospital  Community | CBI | 40.24±15.40 | 7 |
| **Xing Wu(33)**  **2015** | cross-sectional study | 101 | Spouses (17.82%)  Children (72.28%)  Other relatives (9.90%) | 42/59(41.6%) | Hospital | ZBI | 33.04±19.622 | 6 |
| **Jianjun Yin(34)**  **2018** | cross-sectional study | 335 | Spouses (44.18%)  Children (45.67%)  Other relatives (10.45%) | Not mentioned | Hospital | CBI  FBS | CBI56.8±17.3  FBS17.9±8.7 | 6 |
| **Meili Yang(35)**  **2019** | cross-sectional study | 94 | Spouses (36.17%)  Children (39.36%)  Other relatives (24.47%) | Not mentioned | Community | CBI | Not mentioned | 7 |
| **Masami Fukuda(36)**  **2020** | cross-sectional study | 294 | Spouses (42.2%)  Children (52.4%)  Other relatives (5.4%) | Not mentioned | Hospital | CBI | Not mentioned | 8 |
| **Fang Jin(37)**  **2020** | cross-sectional study | 160 | Spouses (21.88%)  Children (48.75%)  Other relatives (29.37%) | 107/53(66.9%) | Hospital  Community | CBI | 53.32±13.398 | 7 |
| **Jun Jin(38)**  **2021** | cross-sectional study | 176 | Spouses (52.27%)  Children (35.80%)  Other relatives (11.93%) | 102/74(58%) | Hospital | CBI | 53. 89±11. 27 | 6 |
| **Yan Zhang(39)**  **2021** | cross-sectional study | 105 | Not mentioned | Not mentioned | Hospital | CBI | 44.33±20.13 | 5 |

Note: CBI: Caregiver Burden Inventory, ZBI: Zarit Burden Interview, FBS: Family Burden Scale, PSS: Perceived Stress Scale
